# How to Address Non-normality: A Taxonomy of Approaches, Reviewed, and Illustrated

**DOI:** 10.3389/fpsyg.2018.02104

**Published:** 2018-11-06

**Authors:** Jolynn Pek, Octavia Wong, Augustine C. M. Wong

**Affiliations:** ^1^Psychology, The Ohio State University, Columbus, OH, United States; ^2^Kinesiology and Health Sciences, York University, Toronto, ON, Canada; ^3^Mathematics and Statistics, York University, Toronto, ON, Canada

**Keywords:** linear model, non-normality, sandwich estimators, bootstrap, robust statistics, transformation, best practice

## Abstract

The linear model often serves as a starting point for applying statistics in psychology. Often, formal training beyond the linear model is limited, creating a potential pedagogical gap because of the pervasiveness of data non-normality. We reviewed 61 recently published undergraduate and graduate textbooks on introductory statistics and the linear model, focusing on their treatment of non-normality. This review identified at least eight distinct methods suggested to address non-normality, which we organize into a new taxonomy according to whether the approach: (a) remains within the linear model, (b) changes the data, and (c) treats normality as informative or as a nuisance. Because textbook coverage of these methods was often cursory, and methodological papers introducing these approaches are usually inaccessible to non-statisticians, this review is designed to be the happy medium. We provide a relatively non-technical review of advanced methods which can address non-normality (and heteroscedasticity), thereby serving a starting point to promote best practice in the application of the linear model. We also present three empirical examples to highlight distinctions between these methods' motivations and results. The paper also reviews the current state of methodological research in addressing non-normality within the linear modeling framework. It is anticipated that our taxonomy will provide a useful overview and starting place for researchers interested in extending their knowledge in approaches developed to address non-normality from the perspective of the linear model.

Psychological science rests on the application of statistical models to data, with the purpose of better understanding and predicting phenomena. Because of its parsimony and well-understood characteristics, the linear model is one of the most popular models employed in the social and behavioral sciences. The multiple linear model (MLR; Cohen et al., 2003) subsumes the *t*-test, and ANOVA as special cases. This set of models are unified under the assumption of *normality*. In practice, however, data is often observed to be non-normal in psychology (Micceri, 1989; Cain et al., 2017) and its allied sciences (Bono et al., 2017), potentially limiting the degree to which linear models can be appropriately fit to data. Stated differently, non-normality is among the most commonly encountered experiences in statistical practice, especially in psychology, possibly inhibiting the utility of popular linear models.

In the long history of statistics, many approaches to address non-normality have been developed. These approaches differ widely in their philosophies, assumptions, and results. The motivating questions behind this review are: How well does current pedagogy of linear models, in the form of textbooks, acknowledge and address non-normality? Which methods for addressing non-normality are popular and valid? When should one method be applied over others? To address these questions, this review surveys, organizes, and describes a large body of methodological research on approaches developed to address non-normality. We emphasize approaches that continue to fall within the linear-modeling framework because researchers already familiar with the linear model can seamlessly incorporate these less well-known advances in practice. By appropriately addressing non-normality, resulting conclusions are more defensible against threats to statistical conclusion validity due to model misspecification (Shadish et al., 2002).

We begin by reviewing the linear model and it assumptions, followed by briefly describing methods developed to address assumption violation, especially normality. Next, we report results of a review of statistics textbooks, primarily used in the behavioral sciences, which were published from 2003 to 2018. We then present a new taxonomy for organizing the many alternative approaches identified in the textbook review according to their underlying motivations and outcomes. We anticipate that this framework will provide a structured overview in terms of how these methods relate to one another, and when should one method be applied over others. Because the treatment of these methods in the reviewed textbooks was often cursory, we also extensively describe each of these methods in a relatively non-technical manner and illustrate their application. Our examples remain within the linear modeling framework, emphasizing situations where distinct results can arise from the same data. Because the choice of one approach over another can change results, we conclude with a general discussion of guidelines for best practice.

## 1. The linear model

The MLR is a general analytic framework where *t*-tests and ANOVA are special cases. For the single case *i*, where *i* = 1, ⋯ , *N*, the linear model is expressed as

(1) $$\begin{array}{l} y_{i}=\beta_{0}+\beta_{1}x_{1i}+\cdots +\beta_{K}x_{Ki}+\epsilon_{i}, \end{array}$$

where *y*_*i*_ is the observed value for the continuous dependent variable (DV) or outcome for case *i*; *x*_*ki*_ is the observed value for case *i* on the *k*th independent variable (IV) or predictor, where *k* = 1, ⋯ , *K*; and ϵ_*i*_ is the unknown error for case *i*. The unknown population model parameters to be estimated are the intercept, β_0_, and the *K* regression slopes, β_1_, ⋯ , β_*K*_. The intercept is interpreted as the expected value of *y* when all *x*_*k*_ = 0; and each *k*th regression slope is the expected conditional change in *y* due to a 1-unit change in *x*_*k*_, holding all other IVs in the model constant.

To obtain sample estimates, $\hat{\beta }={\left({\hat{\beta }}_{0},{\hat{\beta }}_{1},\cdots \;,{\hat{\beta }}_{K}\right)}^{\prime }$, of the unknown parameters, $\beta ={\left(\beta_{0},\beta_{1},\cdots \;,\beta_{K}\right)}^{\prime }$, the ordinary least squares (OLS) criterion is applied where the sum of squared residuals,$\overset{N}{\underset{i=1}{\sum }}e_{i}^{2}$, is minimized. Residuals for each case *i*, *e*_*i*_, serve as estimates of the *N* unknown errors ϵ_*i*_, where $e_{i}=y_{i}-\left[{\hat{\beta }}_{0}+{\hat{\beta }}_{1}x_{1i}+\cdots +{\hat{\beta }}_{K}x_{Ki}\right]$. Obtaining estimates for the parameters in Equation (1) does not require any distributional assumptions.

### 1.1. Assumptions

The linear model has four assumptions. First, the relationship between the IVs and the DV is linear. Second, when inferences about the population parameters are to be made, it is only in the instance when *N* is not large enough that the distributional assumption of normality is placed on the errors [i.e., $\epsilon_{i}\sim N\left(0,\sigma^{2}\right)$, where σ^2^ is the unknown variance of the errors]. Normality of ϵ_*i*_ assures that the sampling distribution of the estimates follow a *t*-distribution when σ is estimated. When *N* is large enough, the sampling distribution will be approximately normal because of the Central Limit Theorem (CLT; described later in more detail). Common inferential devices are null hypothesis significance tests (NHSTs) and their *p*-values, and confidence intervals (CIs), which are probabilistic statements about the unknown population parameters based on sample estimates. Third, σ^2^ is assumed to be finite. Fourth, the *N* cases are assumed to be independently and identically distributed (i.e., no dependencies). Given these assumptions and *N* observations, the vector of errors **ϵ** follow a multivariate normal distribution with mean vector **0**, and a homogeneous variance structure $\Sigma_{\epsilon }=\sigma^{2}I_{N}$, where ***I***_*N*_ is a *N* × *N* identity matrix such that $\epsilon \sim MVN\left(0,\sigma^{2}I_{N}\right)$. For instance, when *N* = 2, $\Sigma_{\epsilon }=\sigma^{2}I_{2}=\left(\begin{array}{cc} \sigma^{2} & 0\\ 0 & \sigma^{2} \end{array}\right)$.

When the assumption of normality is placed on the errors, it is often implicitly assumed that the predictors, $X={\left(x_{i},\cdots \;,x_{K}\right)}^{\prime }$, are fixed (e.g., levels of drug dosage which were randomly assigned). An alternative approach is to consider observed values on the IVs as realizations of random variables (e.g., scores on GPA); this alternative requires the additional assumption that ***X*** is independent of ***ϵ***. It follows then that the errors, ***ϵ***, of the linear model incorporating such IVs would also follow the same normal distribution where the IVs are assumed to be fixed. In brief, either scheme of fixed or random IVs results in essentially equivalent distributional assumptions. Interested readers should refer to Sampson (1974) for nuances between the scheme of fixed vs. random IVs.

In practice, the assumption of normality (and homoscedasticity) of the errors, ***ϵ***, is empirically evaluated by examining the distribution of the residuals, ***e*** (illustrated in the examples to follow). It would be inappropriate to examine the distribution of $y={\left(y_{1},\cdots ,y_{N}\right)}^{\prime }$ independent of the *K* predictors because the distributional assumption is about ***ϵ***. It is only under the intercept-only model, where the distributional properties of ***y*** are identical to ***ϵ***, that in this vein, the distribution of ***y*** is analogous to ***e*** (shown in the first two examples). However, the accuracy of using ***e*** to approximate ***ϵ*** remains to be evaluated.

In the review of textbooks to follow, the following methods were mentioned as approaches that take assumption violation into account: data transformations (i.e., apply a non-linear function to the data); invoking the CLT; rank-based non-parameteric approaches (e.g., the sign test); the bootstrap (i.e., empirically constructing the sampling distribution of estimates); trimming (i.e., removing outliers); Winsorizing (i.e., recoding outliers to less extreme values); heteroscedastic consistent covariance matrices (HCCMs; which allows for heteroscedasticity in place of homoscedasticity); and non-linear models (e.g., logistic regression). Details to these methods, and examples of their application, are provided later in the section on addressing non-normality. SAS and R code showing how these methods can be applied are provided in the Supplemental Material.

## 2. Systematic review of textbooks

### 2.1. Methodology

In this review, we report on the recommendations made in textbooks focused on the linear model when non-normality is encountered in practice. A total of *N* = 61 applied statistics textbooks were identified from several sources: educational publishers' websites (e.g., Pearson Education, Houghton Mifflin Harcourt, Sage Publishers, and Taylor and Francis), Amazon.com, and research university library catalogs. The keywords used in the textbook search are “linear" or “introduction", and “statistics." There were two inclusion criteria: (a) textbooks must have adequate coverage (i.e., several book chapters) of the linear model and its special cases (i.e., *z*-test, *t*-test, ANOVA, correlation, linear regression), and (b) the content must be applied in nature where focus is placed on data analysis and interpretation. Textbooks on research methods associated with the linear model (e.g., Goodwin and Goodwin, 2016; Wilson and Joye, 2016) were excluded from this review.

Each selected textbook was independently coded by a senior and junior coder for the frequency of methods noted as a workaround to non-normality. Both coders are authors of this article and have formal training in quantitative methodology and behavioral statistics. The frequency of suggested methods, which are objective observations, were double coded to avoid data entry errors. All disagreements were cross-checked and verified by referring back to the relevant textbook entry, resulting in a single and accurate data set for analysis. This coding methodology is consistent with similar reviews (e.g., Weidman et al., 2017). Note that only the latest available edition of a textbook was included in the review; textbooks with distinct titles but written by the same authors were included without corrections to content dependency (e.g., *Statistics for the Behavioral and Social Sciences: A Brief Course* by Aron et al., 2010 and *Statistics for Psychology* by Aron et al., 2012).

### 2.2. Results and discussion

Table 1 presents results of the review, stratified by graduate vs. undergraduate textbooks. The presentation of the methods are ordered according to how often they were suggested in graduate textbooks, with data transformations being mentioned most often (89%), followed by an argument of robustness of the results due to the CLT (56%) Next, rank-based non-parameteric methods (50%) and the bootstrap (50%) were equally suggested, followed by less popular methods. Note that the method of applying a reverse transformation is only pertinent to the method of data transformations; given that data transformations were recommended, reverse transformations were also suggested 19% of the time. Reverse transformations are also called back transformations. Let ***y*** represent an observed variable, which is non-normally distributed, and *g*(·) be a transformation such that *g*(***y***) follows a normal distribution. The reverse of the transformation, *g*(·), is denoted by *g*^−1^(·). As an example, *g*(·) can be log(·) such that the reverse transformation, *g*^−1^(·), is exp(·). Mathematically expressed, *g*(*y*) = log(*y*) = *w*; then *g*^−1^(*w*) = exp(*w*) = *y*.

**Table 1:** Frequency and counts of approaches for addressing non-normality across statistics textbooks published from 2003 to 2018.

	**Graduate** (***n*** = **18**)		**Undergraduate** (***n*** = **43**)	
	**n**	**%**	**n**	**%**
Transform	16	89	17	38
Reverse transform	3	17	2	4
CLT	10	56	35	78
Rank-based Nonparametric	9	50	34	76
Bootstrap	9	50	6	13
Trim	6	33	9	20
Winsorize	5	28	4	9
HCCM	3	17	0	0
Nonlinear models	3	17	0	0
Not covered	1	6	3	7

*Methods are ranked ordered according to the most popular method mentioned in graduate textbooks. CLT, central limit theorem; HCCM, heteroscedasticity-corrected covariance matrix. Not covered implies that even the CLT was not mentioned. The percentages reported do not sum to 100% because textbooks can include more than one method for addressing non-normality*.

The rank order of suggested methods was different between graduate vs. undergraduate textbooks. Instead of most often recommending data transformations, undergraduate textbooks emphasized robustness due to the CLT (78%) as well as the use of rank-based non-parametric counterparts (76%). This trend is unsurprising because the CLT does not require changing the usual data analytic approach within the linear modeling framework. Most undergraduate textbooks also introduced rank-based non-parametric methods in later chapters, justifying their recommendation in earlier chapters focused on the linear model. Transformations, including reverse transformations (12% of instances where transformations were suggested), was recommended 38% of the time followed by the less popular methods. Though limited in frequency of being recommended, reverse transformations require careful implementation to obtain accurate results (Duan, 1983; Zhou and Gao, 1997; Pek et al., 2017a,b).

Trimming is recommended more often than Winsorizing (33 vs. 28% among graduate textbooks, and 20 vs. 9% among undergraduate textbooks). Finally, it is of concern that a small but non-zero proportion of textbooks did not address non-normality. Conversely, it was a positive note that a larger but limited number of textbooks recommended non-linear models.

To provide guidance regarding these methods, we detail underlying causes of non-normality below. Then, each suggested method from Table 1 is described in relation to the assumptions of the linear model, and classified according to a taxonomy organized by three characteristics: (a) remain within the linear modeling framework, (b) change the nature of the data, and (c) treat non-normality either as a nuisance or an important aspect of the data (see Table 2).

**Table 2:** Taxonomy of methods developed to address non-normality.

**Method**	**Linear model**	**Keep data as is**	**Non-normality is informative**
CLT	✓	✓	✗
HCCM	✓	✓	✗
Bootstrap	✓	✓	✗
Trim or Winsorize	✓	✗	✓^†^
Transform	✓	✗	Depends
Rank-based Nonparametric	✗	✗^‡^	✗
Nonlinear models	✗	✓	✗

CLT, central limit theorem; HCCM, heteroscedasticity-corrected covariance matrix.

† Trimming and Winsorizing treat non-normality as an indication of contamination by outliers; the outliers are themselves treated as nuisance.

‡ *Rank-based nonparametric approaches tend to focus on the rank order in the data by ignoring any quantitative information; technically, instead of transforming the data, order statistics (e.g., minimum and maximum observations) are computed to take the place of usual sufficient statistics (e.g., mean and variance)*.

## 3. Addressing non-normality (and heteroscedasticity)

Recall that the assumption of normality can be relaxed when sample size *N* is large enough; the errors need not follow a normal distribution because of the CLT. Regardless of the distribution of ***ϵ***, the CLT assures that the sampling distribution of the estimates will converge toward a normal distribution as *N* increases to infinity, when ***ϵ*** are independent and identically distributed, and when σ^2^ is finite. Stated differently, the assumption of normality is *inessential* with large enough *N*. By employing the CLT, inference should technically be based on the *z*-distribution instead the *t*-distribution. One practical question is, how large should *N* be such that the CLT can be reasonably invoked? For the limited case of a DV without IVs, the reviewed textbooks have suggested a range of *N* ≥ 15 (e.g., Jaccard and Becker, 2009) to *N* ≥ 50 (e.g., Hanna and Dempster, 2013). Such rules of thumb tend to be inaccurate because the size of *N* for the CLT to be in place is a function of the number of *K* IVs and the extent of non-normality of the errors (e.g., see Pek et al., 2017b). In general, larger *N* is required when the errors depart more from normality; specifically, convergence due to the CLT is faster when errors are symmetric in distribution (i.e., less skewed; Lange et al., 1989; Pek et al., 2017b).

When non-normality in ***e*** is observed, two assumptions in the linear model are potentially unmet. First, non-normality in ***e*** suggests non-normality in ***ϵ*** (i.e., the assumed structure of ***ϵ*** is misspecified), which results in inaccurate inferential results regarding *p*-values and CI coverage. Second, the relationship between ***X*** and ***y*** may not be linear, and the misfit could be observed from non-normal residuals. Additionally, if the unknown population functional form between ***X*** and ***y*** is non-linear and a linear model is fit, instead, the estimates of the linear model are biased estimates of the unknown population parameters. Stated succinctly, the observed non-normality in ***e*** may indicate model misspecification in terms of the linear relationship between ***X*** and ***y***.

Violating the assumption of normal ***ϵ*** is, however, not necessarily fatal when sample size, *N*, is large enough for the CLT to be at work. Besides invoking the argument of robustness of model results due to the CLT, several other methods have been suggested among the 61 reviewed textbooks to take into account non-normality of observed ***e***. These methods are classified in Table 2 according to whether they remain within the linear modeling framework, modify the data, and treat the presence of non-normality as informative or a nuisance. In general, other than the CLT and bootstrap, methods which remain within the linear modeling framework are implicitly small sample alternatives.

The methods listed in Table 2 are rank ordered according to how much they depart from OLS regression (i.e., CLT). The CLT relies on the robustness of the solution when *N* is large, requiring no changes in the application of the linear model to data. Using heteroscedasticity-corrected covariance matrices (HCCM) or the bootstrap changes only the estimator in terms of determining the sampling distribution of the estimates. Trimming and Winsorizing involve changes to the data, by removing or modifying outliers, which necessitates a change in the estimator although the linear model continues to be applied to the data. Depending on the transformation used, non-normality is either treated as a nuisance or informative. When rank-based non-parametric and non-linear models are applied to data, the linear model is abandoned. Rank-based non-parametric methods circumvent the issue of non-normality of the residuals by analyzing ranks of the data. In non-linear models, the non-normality in the residuals are explicitly modeled. Below, we detail these methods, and illustrate the utility of methods, which remain within the linear model, with several empirical examples.

### 3.1. Heteroscedastic corrected covariance matrix (HCCM)

As nomenclature suggests, the method of HCCMs was developed to specifically address violation of the homoscedastic distributional assumption (i.e., the covariance structure of the errors, $\Sigma_{\epsilon }=\sigma^{2}I_{N}$), and *not* that of normality. Very often, however, HCCMs are applied in practice to address general forms of misspecification including non-normality (Dudgeon, 2017). We thus review HCCMs for completeness, and clarify that HCCMs do not take into account non-normality. All three graduate textbooks which mention HCCM (see Table 1) correctly identify the method as an approach to address heteroscedasticity. By employing this approach to address observed heteroscedasticity in ***e***, the user implicitly assumes that model misspecification is in the covariance structure of ***ϵ***. By contrast, the functional form relating ***X*** to ***y*** is assumed to be correct. Here, heteroscedasticity of unknown form in ***ϵ***, which is estimated by ***e***, is regarded as a nuisance to be addressed. Note that ***ϵ*** can still follow a multivariate normal distribution but be heteroscedastic. Given *N* = 2, an example of a normal but heteroscedastic covariance structure is $\Sigma_{\epsilon }=\left(\begin{array}{cc} \sigma_{1}^{2} & 0\\ 0 & \sigma_{2}^{2} \end{array}\right)$, where $\sigma_{1}^{2}\neq \sigma_{2}^{2}$.

Given homoscedasticity and the Gauss Markov theorem, OLS is the best linear unbiased estimator (BLUE) for the linear model, and $\hat{\beta }={\left(X^{\prime }X\right)}^{-1}X^{\prime }y$. Further, the asymptotic covariance matrix of $\hat{\beta }$, $\Sigma_{\hat{\beta }}={\left(X^{\prime }X\right)}^{-1}X^{\prime }\Sigma_{\epsilon }X{\left(X^{\prime }X\right)}^{-1}$ (Hayes and Cai, 2007), reduces to σ^2^(***X***′***X***)^−1^ because $\Sigma_{\epsilon }=\sigma^{2}I_{N}$. Here, σ^2^ is estimated by the mean squared residual, ${\hat{\sigma }}^{2}=\overset{N}{\underset{i=1}{\sum }}e_{i}^{2}/df$, where *df* = (*N* − *K*) is the degrees of freedom. Standard errors of $\hat{\beta }$ are the square root of the diagonal elements of $\Sigma_{\hat{\beta }}$. When homoscedasticity is violated, $\hat{\beta }$ remains unbiased but *p*-values reflecting NHSTs and CI coverage about ***β*** will be incorrect (Long and Ervin, 2000), because $\hat{\beta }$ no longer retains the property of BLUE. As such, $\hat{\beta }$ will not have the smallest variance among all the linear unbiased estimators of ***β***. When *N* is not large, assuming homogenous variance in the presence of heteroscedasticity can result in either conservative or liberal NHSTs and improper CI coverage.

The development of HCCMs can be traced to Eicker (1963, 1967) and Huber (1967). Later, White (1980), MacKinnon et al. (1985), and Davidson and MacKinnon (1993) formalized the form of the HCCM known as HC0 and presented three alternatives for small sample conditions called HC1 (derived by Hinkley, 1977), HC2, and HC3. More recently, Cribari-Neto and colleagues developed further modifications called HC4 Cribari-Neto (2004), HC4M Cribari-Neto and da Silva (2011), and HC5 Cribari-Neto et al. (2007). To date, there are at least seven versions of HCCMs which are asymptotically equivalent. For more extensive reviews of this work, see Long and Ervin (2000), Hayes and Cai (2007), and Dudgeon (2017). Below, we highlight the rationale behind HC0, HC1, HC2, and HC3.

Note that HC0 is also called the Huber-White estimator of $\Sigma_{\hat{\beta }}$:

$$\begin{array}{l} \text{HC0}={\left(X^{\prime }X\right)}^{-1}X^{\prime }\text{diag}\left[e_{i}^{2}\right]X{\left(X^{\prime }X\right)}^{-1}, \end{array}$$

where $diag\left[e_{i}^{2}\right]$ is a *N* × *N* diagonal matrix of the squared residuals. When *N* = 2, $\text{diag}\left[e_{i}^{2}\right]=\left(\begin{array}{cc} e_{1}^{2} & 0\\ 0 & e_{2}^{2} \end{array}\right)$. Instead of assuming homoscedasticity of **Σ**_***ϵ***_, the squared residuals, $e_{i}^{2}$, are employed as estimators of the variance of ϵ_*i*_. Such estimators have come to be known as *sandwich estimators*, because they follow a form where two slices of “bread” (e.g., (***X***′***X***)^−1^***X***′ and ***X***(***X***′***X***)^−1^) envelope a middle (e.g., $\text{diag}\left[e_{i}^{2}\right]$). The remaining versions of HCCMs make modifications to HC0, often to the matrix in the middle.

$$\begin{array}{l} \text{HC1}=\frac{N}{df}\text{HC0} \end{array}$$

incorporates a degree of freedom correction by scaling each *e*_*i*_ by a factor of $\sqrt{N/df}$.

The motivation for HC2 takes into account the effect of observations with high leverage. Leverage values quantify the extent to which a case's IV values lie away from the centroid of the other cases' IV values. The hat matrix in the linear model ***H*** = ***X***(***X***′***X***)^−1^***X***′ maps the DV vector, ***y***, to the vector of fitted or predicted values, $\hat{y}=X\hat{\beta }$, where $\hat{\beta }=Hy$. The diagonal elements of the *N* × *N* hat matrix, ***H***, are leverage values, $h_{ii}=x_{i}{\left(X^{\prime }X\right)}^{-1}x_{i}^{\prime }$ where ***x***_*i*_ is the vector of IVs for the *i*th case. Higher values on *h*_*ii*_ indicate larger extremity of ***x***_*i*_ from the centroid of ***X***. The variance of *e*_*i*_ is $\sigma^{2}\left(1-h_{ii}\right)$, implying that $e_{i}^{2}/\left(1-h_{ii}\right)$ is a less biased estimator of the variance of ϵ_*i*_ compared to $e_{i}^{2}$ (see Long and Ervin, 2000). Taken together,

$$\begin{array}{l} \text{HC2}={\left(X^{\prime }X\right)}^{-1}X^{\prime }\text{diag}\left[\frac{e_{i}^{2}}{1-h_{ii}}\right]X{\left(X^{\prime }X\right)}^{-1}. \end{array}$$

Note that *h*_*ii*_ ≤ 1, and the weight $\frac{1}{1-h_{ii}}$ increases the influence of high leverage cases in the computation of HC2. For example, when *h*_*ii*_ = 0.9, $\frac{1}{1-h_{ii}}=10$, whereas when *h*_*ii*_ = 0.2, $\frac{1}{1-h_{ii}}=1.25$. As an extension to HC2 and an approximation of a jackknife estimator of Efron (1982, cited in MacKinnon et al., 1985),

$$\begin{array}{l} \text{HC3}={\left(X^{\prime }X\right)}^{-1}X^{\prime }\text{diag}\left[\frac{e_{i}^{2}}{{\left(1-h_{ii}\right)}^{2}}\right]X{\left(X^{\prime }X\right)}^{-1}. \end{array}$$

The weight $\frac{1}{1-h_{ii}}$ is modified to $\frac{1}{{\left(1-h_{ii}\right)}^{2}}$, such that the effect of leverage is further inflated in HC3 relative to HC2. For example, when *h*_*ii*_ = 0.9, $\frac{1}{{\left(1-h_{ii}\right)}^{2}}=100$ whereas when *h*_*ii*_ = 0.2, $\frac{1}{{\left(1-h_{ii}\right)}^{2}}=1.5625$. HC4, HC4M, and HC5 are extensions of HC3 where the weight is modified by its power value, λ: $\frac{1}{{\left(1-h_{ii}\right)}^{\lambda }}$. Note that HC3 is defined by λ = 2. Among the HCCMs reviewed here, HC3 is recommended in samples of *N* ≤ 250 (Long and Ervin, 2000).

#### 3.1.1. Example 1: daily newspaper reading

A small data set on newspaper reading habits of persons from *N* = 14 European Union countries, reported in De Veaux et al. (2015, p. 695), is employed to illustrate the utility of HCCMs in the presence of non-normal residuals. These data were collected by Eurostat, the statistical office of the European Union. Of interest was any sex difference in adult lifelong learning. Here, 1000 respondents from each European country provided data, and percentages of males' and females' daily reading of newspapers were analyzed. The potential sex difference was operationalized as the (arithmetic) mean of male minus female percentages, and a paired samples *t*-test is planned. The linear model is *y*_*i*_ = β_0_ + ϵ_*i*_, where *y*_*i*_ is a country's sex difference in percentage points, and ${\hat{\beta }}_{0}$ is the estimated mean sex difference.

**Figure 1:**
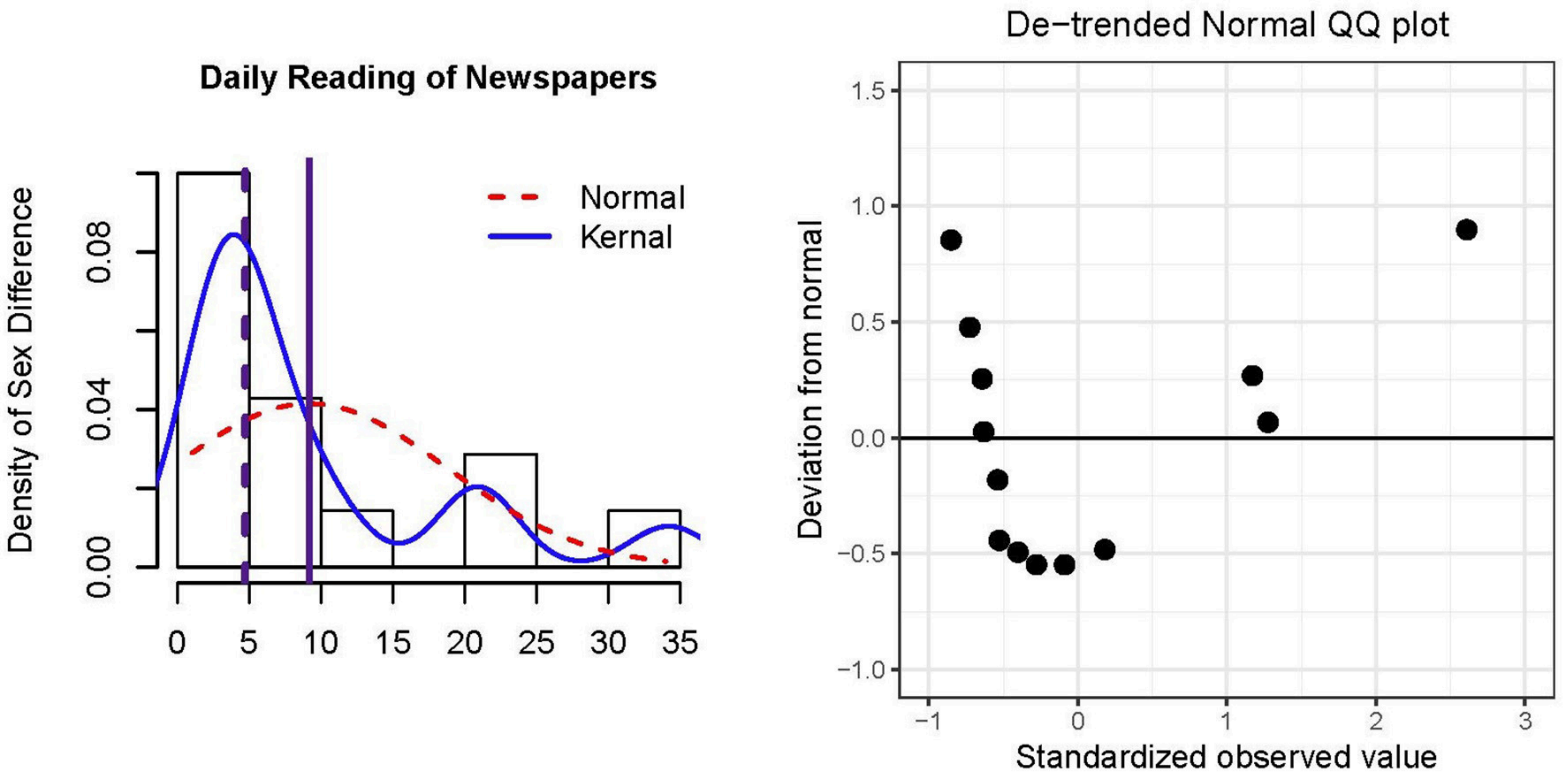
Histogram and de-trended QQ plot of residuals of *N* = 14 European countries' percentage differences in daily newspaper reading for males minus females. The solid vertical reference line in the histogram represents the mean, and the dashed vertical reference line represents the median.

Figure 1 presents a histogram of the data and residuals, which are equivalent in an intercept-only model. The normal and kernel distributions are overlaid by dashed and solid lines, respectively. In general, a majority of the countries did not have large sex differences in daily newspaper reading (≤ 5% difference), although there was a noticeable positive skew (*skewness* = 1.74) and positive excess *kurtosis* = 2.54. Unsurprisingly, the *mean* = 9.17 (solid vertical line) is pulled toward the tail in comparison to the *median* = 4.70 (dashed vertical line). The positive mean and median indicate that a larger percentage of males relative to females read newspapers daily. The right panel of Figure 1 is a de-trended QQplot (Thode, 2002, p. 25) of the residuals, where the horizontal line represents quantiles of a normal distribution and the points represent quantiles of the kernel distribution. The de-trended version removes potential visual bias due to orthogonal distances between the QQpoints and the 45° reference line in the usual QQplot. The distribution of residuals largely departs from normality as the points do not lie closely to the reference line.

Table 3 presents inferential information regarding sex differences in reading the newspaper daily. 9.17% of males more than females reported reading the newspaper daily. This estimated mean difference was significant for the CLT approach as well as the four HCCMs; and there was some variability across these results because of small *N*. If the median was used in place of the mean, the sex difference in daily newspaper reading would be smaller in magnitude. Recall that HCCMs were developed for small samples for two reasons. First, the effect of each case's leverage increases with smaller *N*, resulting in larger distinctions among the HCCMs. Second, with increasing *N*, the sampling distribution of $\hat{\beta }$ will approach normality with the CLT such that alternative methods need not be employed to address non-normal residuals, ***e***. In the next example, where *N* is large, differences in the results among the CLT and HCCM methods are minimal.

**Table 3:** Sex difference in percentages for examples 1 and 2.

**Method**	**Daily newspaper reading** (***N*** = **14**)					**Primary school enrollment** (***N*** = **117**)				
	**$\hat{\beta }$**	***S.E.***	***t***	***p***-**value**	**95%** ***CI****s*	$\hat{\beta }$	***S.E.***	***t***	***p***-**value**	***95%*** ***CI*s**
CLT	9.17	2.57	3.58	0.0034	[3.63, 14.71]	1.03	0.36	2.86	0.0050	[0.32, 1.75]
HC0	9.17	2.47	3.71	0.0026	[3.83, 14.51]	1.03	0.36	2.87	0.0048	[0.32, 1.75]
HC1	9.17	2.57	3.58	0.0034	[3.63, 14.71]	1.03	0.36	2.86	0.0050	[0.32, 1.75]
HC2	9.17	2.57	3.58	0.0034	[3.63, 14.71]	1.03	0.36	2.86	0.0050	[0.32, 1.75]
HC3	9.17	2.66	3.45	0.0043	[3.42, 14.92]	1.03	0.36	2.85	0.0052	[0.32, 1.75]
percBS	9.17	–	–	–	[4.55, 13.80]	1.03	–	–	–	[0.35, 1.77]
BCa	9.17	–	–	–	[5.21, 15.02]	1.03	–	–	–	[0.37, 1.81]
Winsorize	6.30	1.66	3.79	0.0068	[2.37, 10.23]	0.35	0.18	1.91	0.0601	[–0.015,0.72]
Trim	5.76	1.61	3.56	0.0090	[1.96, 9.59]	0.25	0.18	1.35	0.1820	[–0.12, 0.61]

*S.E., standard error; CI, confidence interval; CLT, central limit theorem; HC, heteroscedastic consistent method; percBS, percentile bootstrap; BCa, bias corrected and accelerated bootstrap. Estimates are in the direction of male percentages minus female percentages. Winsorized and trimmed means pertain to modifying about 20% of the tail distributions; 20.43% for Example 1 and 20.51% for Example 2*.

#### 3.1.2. Example 2 primary school enrollment

The World Health Organization (WHO) compiles health statistics for its member states. As an indicator of primary school accessibility, the net primary school enrollment percentage was collected for males and females for *N* = 117 countries. These data for 2006 are reproduced in De Veaux et al. (2015, p. 695–696). Of interest is whether there are gender disparities in accessibility to basic education. Similar to Example 1, the expected sex difference is operationalized as a mean of male minus female percentages in primary school enrollment.

**Figure 2:**
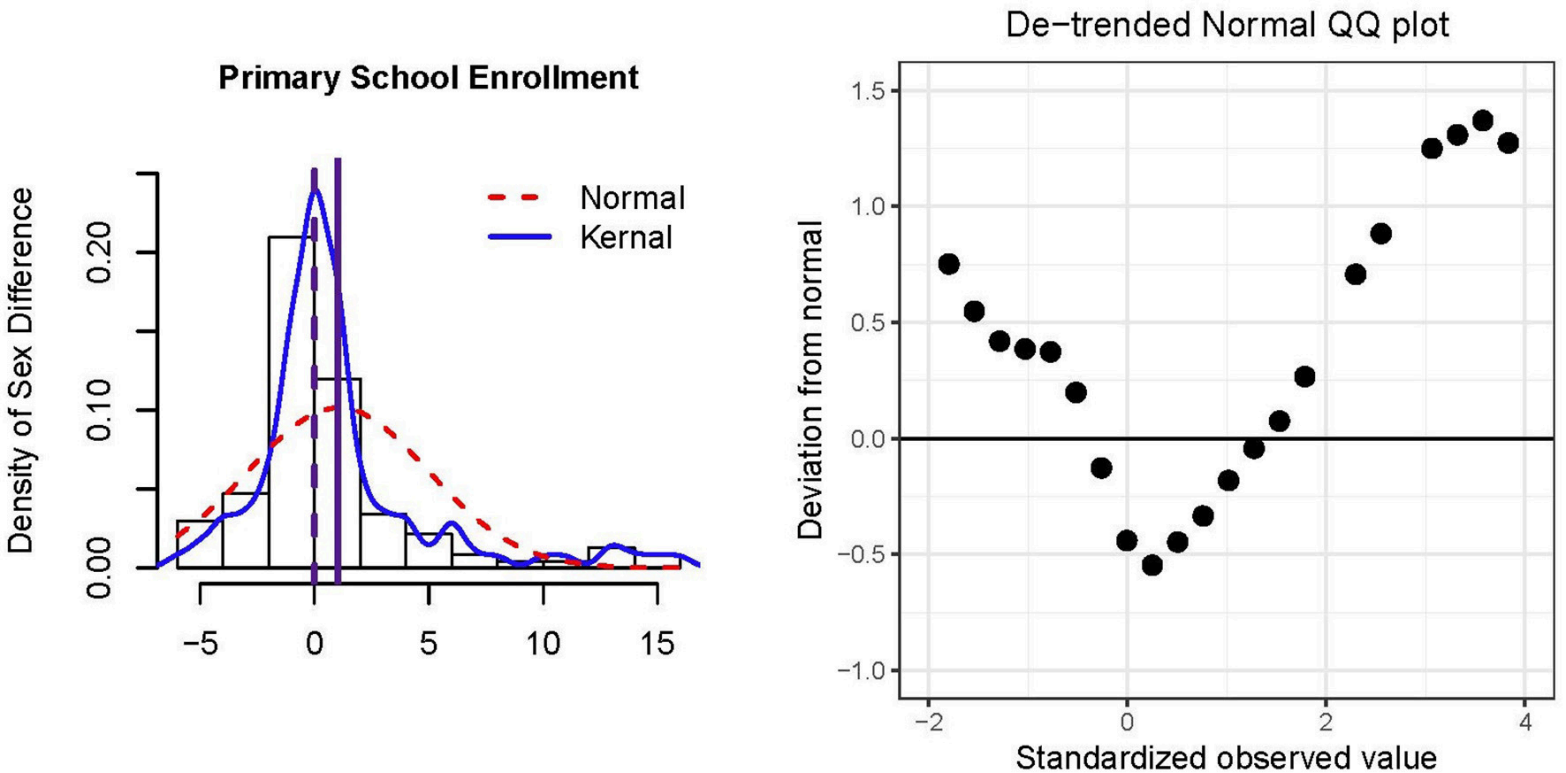
Histogram and de-trended QQ plot of residuals of *N* = 117 countries' percentage differences primary school enrollment for males minus females. The solid vertical reference line in the histogram represents the mean, and the dashed vertical reference line represents the median.

Figure 2 presents a histogram and de-trended QQ plot of the residuals and data. The majority of countries did not show gender disparity in primary school enrollment although the distribution was not normal (*skewness* = 1.86, excess *kurtosis* = 4.31). Extreme sex differences tended to be in favor of males having more access to primary education compared to females, resulting in the positive skew. However, a larger percentage of small differences favored females over males such that the *median* = 0 was smaller than the *mean* = 1.03. From Table 3, there was a significant sex difference in percentage of enrollment in primary school education; 1.03% of males received primary education more than that of females, and this result is significant across the CLT and HCCM methods. Indeed, because *N* = 117 is relatively large, there is limited variability among these methods (cf., Example 1). If the median was used to operationalize sex differences, conclusions would be qualitatively different from that of the mean (e.g., see results for Winsorizing and trimming in Table 3).

### 3.2. Bootstrap

The family of bootstrap methods (see Efron and Tibshirani, 1993 for a good review) is similar to the family of HCCMs in that the linear model continues to be fit to the data; the data are not altered; and non-normality in the residuals, ***e***, are treated as non-informative and a nuisance to be addressed. As noted above, the bootstrap is loosely related to HCCMs in that the HC3 estimator is an approximation of the jackknife (Efron, 1982, cited in MacKinnon et al., 1985); the jackknife is also an approximation of the bootstrap (Efron, 1979). In contrast to HCCMs, the bootstrap does not make any assumptions regarding the sampling distribution of $\hat{\beta }$ or of the errors, ***ϵ***. Instead, the bootstrap rests on the less restrictive assumption of the sample being representative of the population, making it a large sample method akin to the CLT (cf., HCCMs which are a small sample method). A small sample, by definition, cannot be representative of the population. With this assumption, the sampling distribution of $\hat{\beta }$ is empirically constructed via a computationally intensive method as outlined below.

To bootstrap the sampling distribution of $\hat{\beta }$, the sample of size *N* is treated as a surrogate to the population of interest. Next, *B* bootstrap replicates of size *N* are drawn from the original sample, or surrogate population, with replacement. The linear model is then fit to each *b* = 1, ⋯ , *B* replicate and ${\hat{\beta }}_{b}$ is computed. This process of sampling from the original data empirically simulates random sampling from the population, and computing *B* sets of ${\hat{\beta }}_{b}$ results in an empirically constructed sampling distribution of $\hat{\beta }$. According to Fox (2008, p. 590), “The population is to the sample as the sample is to the bootstrap samples.” Typically, *B* = 1000 or *B* = 5000 such that the sampling distribution, especially its tails, are well-approximated.

**Figure 3:**
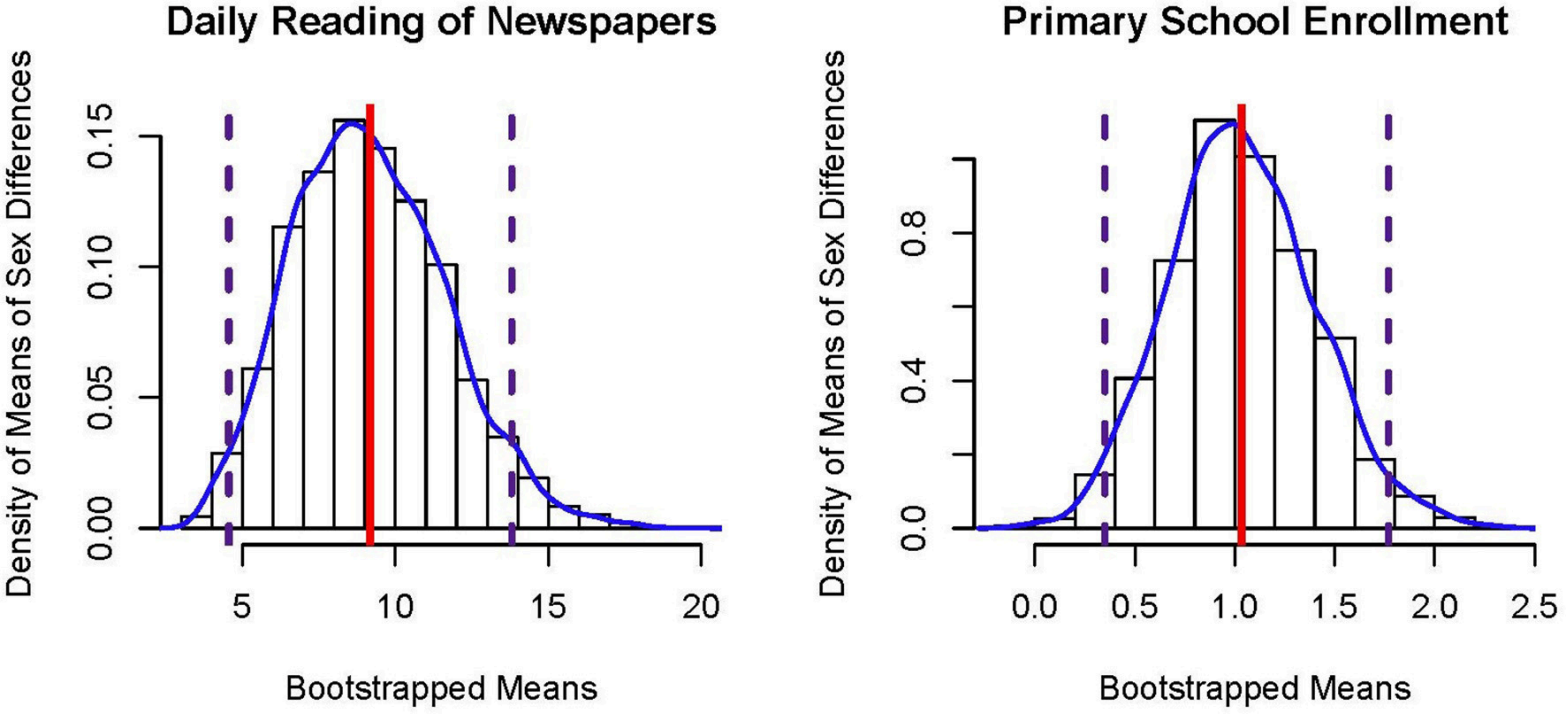
Histograms of bootstrapped sampling distributions of the mean of sex differences in percentages. The solid vertical line represents the estimate, $\hat{\beta }$, and the dashed vertical lines represent lower and upper bounds to the 95% percentile bootstrap CI.

The empirical distribution constructed by the *B* bootstrapped estimates will typically be asymmetric and thus non-normal. Figure 3 presents bootstrap sampling distributions of the mean sex difference in reading newspapers daily (left panel) and primary school enrollment (right panel), which are both positively skewed. Although bootstrap *p*-values derived from standard error estimates (which assume a symmetric sampling distribution) can be computed, non-parametric bootstrap confidence intervals based directly on such empirically constructed sampling distributions are more often employed. Note that there is much more observed variability (cf., ${\hat{\sigma }}^{2}/N$) in the bootstrapped means for sex differences in daily newspaper reading compared to primary school enrollment due to differences in sample sizes; *N* = 14 vs. *N* = 117, respectively.

The (1−α)100% percentile bootstrap is constructed where the lower and upper bounds of the CI are defined by the $\left(\frac{\alpha }{2}\right)100$th and $\left(1-\frac{\alpha }{2}\right)100$th percentile of this empirically constructed distribution, respectively. For instance, when *B* = 1000, the lower and upper bounds of the 95% percentile bootstrap CI are the 25th and 975th ordered bootstrapped estimates, ${\hat{\beta }}_{b}$, respectively. Small sample improvements to the percentile bootstrap CI have been developed (Efron, 1981, 1987), and a popular version is the bias-corrected, accelerated (BCa) bootstrap CI. See Efron and Tibshirani (1993, chapter 14) for technical details.

Table 3 presents percentile and BCa bootstrap CIs for Examples 1 and 2, where *B* = 1000. In comparison to the CIs obtained via the CLT and HCCM methods, the bootstrapped CIs are asymmetric about the estimate, $\hat{\beta }$. Across these types of approaches, all conclude that there is a sex difference in the two examples. Note that the parameter of interest among these approaches is the arithmetic mean of the non-normally distributed data. In the next set of approaches, the parameter of interest is no longer the arithmetic mean, but a different parameter representing central tendency.

### 3.3. Trimming and winsorizing (robust regression)

Unlike the CLT, HCCM, and bootstrap approaches, trimming and Winsorizing implicitly assume that the observed data are contaminated by the presence of outliers, which are recognized as extreme cases in the tails of the data distribution (e.g., see Tukey and McLaughlin, 1963; Wilcox, 2017). Barnett and Lewis (1998) provide an extensive treatment of outliers; and recent work has focused on their automatic detection (e.g., see Mavridis and Moustaki, 2008; Marmolejo-Ramos et al., 2015). Stated differently, the use of either trimming or Winsorizing presumes that non-normality is due to the presence of improper data, and these erroneous data are discarded or modified. Consider the ordered data of Example 1 on percentage difference between the sexes in daily newspaper reading among *N* = 14 European countries:

$$\begin{array}{l} \begin{array}{cccccccccccccc} 1.0 & 2.2 & 3.0 & 3.1 & 4.0 & 4.0 & 4.1 & 5.3 & 6.5 & 8.3 & 10.9 & 20.4 & 21.4 & 34.2. \end{array} \end{array}$$

Winsorizing involves replacing the extreme values, assumed to be contaminants, to specified percentiles of the data; with 20% Winsorizing, 60% of the data remains unchanged whereas the observations below the 20th and 80th percentile will be limited to observed values of these specific percentiles. The data from Example 1 are transformed with 21.43% Winsorizing below, where the data for three cases at each tail are modified, because each case makes up 7.14% of the data:

$$\begin{array}{l} \begin{array}{cccccccccccccc} 3.1 & 3.1 & 3.1 & 3.1 & 4.0 & 4.0 & 4.1 & 5.3 & 6.5 & 8.3 & 10.9 & 10.9 & 10.9 & 20.9. \end{array} \end{array}$$

In a similar manner, trimming involves excluding extreme cases from the data. Below, data from Example 1 has undergone 21.43% trimming, leaving 57.14% of the data:

$$\begin{array}{l} \begin{array}{cccccccc} 3.1 & 4.0 & 4.0 & 4.1 & 5.3 & 6.5 & 8.3 & 10.9. \end{array} \end{array}$$

Means of the Winsorized or trimmed data are said to be *robust* alternatives to the arithmetic mean in that these modified means are insensitive to extreme values, which have either been transformed or discarded, respectively. Importantly, Winsorizing or trimming the data replaces the population parameter associated with the original data (e.g., the arithmetic mean) with a different parameter of location about the modified data (e.g., the median with 50% trimming). Note that when extreme cases are *not* outliers, but legitimately part of the population, Winsorized or trimmed means are biased estimates of the arithmetic mean. Reflective of the bias-variance tradeoff, Winsorizing and trimming typically yields more powerful NHSTs and tighter CIs about the robustified estimate.

The process of Winsorizing or trimming the data follows from ordering the data such that extreme data points are replaced by less extreme points or are removed. This ordering creates dependency among the data points, violating the assumption of independence required for OLS estimation. To address non-independence, a family of robust location estimators called *M-estimators* have been developed, where “M" stands for “maximum likelihood type." Instead of minimizing the sum of squared residuals under OLS, $\overset{N}{\underset{i=1}{\sum }}e_{i}^{2}$, a different objective function is minimized. The derivative of a function of residuals, denoted by ψ(*e*), is set to 0 in order for it to be minimized; for OLS, ψ(*e*) = 2*e*. As an example of an M-estimator, the Huber (1964) weights or objective function is:

$$\begin{array}{l} \psi \left(e\right)=\{\begin{array}{cc} e & \text{for}|e|\leq k\\ \text{sign(}e\text{)}k & \text{for}|e|>k, \end{array} \end{array}$$

where *k* is the bending constant which demarcates the center of the data distribution from the tails. This Huber (1964) estimator behaves like OLS at the center of the data (i.e., |*e*| ≤ *k*), and like the least absolute values at the tails (i.e., |*e*| > *k*); *k* is determined in part by the extent of Winsorizing or trimming. There are several other robust regression estimators associated with Winsorizing and trimming such as the biweight or bisquare estimator (Beaton and Tukey, 1974) and the least trimmed squares (LTS; Rousseeuw, 1984). Notice that M-estimators are related to HCCMs in that different weights are assigned to cases according to their extremity (e.g., *e*_*i*_ vs. *h*_*ii*_) relative to other cases in the data set. Recently, a generalization of M-estimators was developed in the neural network literature (e.g., Xia and Wang, 2018). For a more detailed treatment of robust estimators, see Wilcox (2017). Although not extensively covered in this review, HCCMs and M-estimators are also related to weighted least squares (WLS) and generalized least squares (GLS) estimators. WLS and GLS are distinct from HCCMs and M-estimators because they require specification of the functional form of the non-normal residuals (e.g., logarithmic; see section 3.5.2 on non-linear models).

Table 3 presents estimates of the Winsorized and trimmed means for Examples 1 and 2, as well as their inferential information. Estimated Winsorized and trimmed mean percentages of the sex difference in both examples, $\hat{\beta }$, are highly distinct from the arithmetic means associated with the other methods, highlighting the change in the nature of the population parameter of interest. Additionally, the standard error estimates were much smaller for the Winsorized and trimmed means relative to the CLT and HCCM methods, illustrating the gain in statistical efficiency. For the newspaper example, all methods led to the same conclusion that a small but statistically significant percentage of males read newspapers daily more often than females.

For the second example, different conclusions are reached when the arithmetic mean vs. the Winsorized or trimmed means are employed to operationalize sex differences in accessibility to primary school enrollment. A significant sex difference in favor of males relative to females is concluded when the CLT, HCCM, and bootstrap methods are applied to the non-normal data. Conversely, under Winsorizing and trimming, there is insufficient evidence to conclude a sex difference; the *t*-tests are non-significant and the 95% CIs include the value β_0_ = 0. Recall that Winsorizing and trimming assume that data at the tails of the distribution are contaminants. Examining the data, countries with large sex differences in favor of males are Pakistan (16%), Central African Republic (15%), Benin (14%), Niger (13%), Mali (13%), Guinea (11%), Burkina Faso (10%), and Djibouti (8%); countries with sex differences in favor of females are Malawi (−6%), Gambia (−5%), Namibia (−5%), Mauritania (−4%), Zambia (−4%), Dominica (−4%), and Armenia (−4%). These data are unlikely to be erroneous to justify the undisputed use of Winsorizing or trimming. Instead, the non-normality and heterogeneity in the data could be informative and reflect unknown but important clusters of countries with distinct characteristics. Such population heterogeneity can be modeled by mixture regression (McLachlan and Peel, 2004) or quantile regression (Koenker and Bassett, 1978; Waldmann, 2018).

### 3.4. Data transformations

The presence of non-normal residuals, ***e***, suggest three scenarios: the errors, ***ϵ***, are non-normally distributed; the functional relationship between ***X*** and ***y*** is non-linear; or both. Because transformations have the potential to address these sources of misspecification while remaining within the linear modeling framework and continuing with OLS estimation, it is unsurprising that transformations is the most often recommended approach in graduate textbooks (see Table 1). A major consequence of using transformations, however, is a change in the scale of the variables (i.e., IVs, DVs, or both), which can often obfuscate interpretation.

Transformations were historically developed to (a) address assumptions of a statistical model, and (b) to aid interpretation (Tukey, 1957). With respect to the linear model, different types of transformations were developed to address different sources of non-normality observed in ***e***. Such non-normality can be treated as a nuisance or be informative and modeled. For instance, (Bartlett, 1947) developed a class of transformations to stabilize the residual variance in ANOVA models. Another example is Mosteller and Tukey's (1977) bulging rule, which recommends certain types of transformations from their family of transformations called the ladder of power to linearize the bivariate relationship between an IV and DV (e.g., see Fox, 2008, chapter 4).

Our brief review of transformations is organized according to whether they are better suited for one of two distinct statistical modeling cultures: prediction vs. inference (see Breiman, 2001 for an overview). Statistical prediction uses the linear model to forecast future outcomes (e.g., market and weather predictions). Conversely, statistical inference places attention on estimating population parameters (e.g., treatment effect or percentage reduction in attempted suicides with a 10 point decrease in depression scores). Our review stems from the inferential perspective, reflecting the statistical culture of the reviewed textbooks save for Harrell (2015). In general, algorithmic or non-parametric transformations are consistent with statistical predictions, which treat non-normality as a nuisance, whereas parametric transformations are consistent with statistical inference, which treat non-normality as informative.^1^ Reverse transformations are pertinent to interpretations made in both prediction and inference.

#### 3.4.1. Algorithmic transformations

Algorithmic transformations are determined from optimizing a function according to certain criteria (e.g., maximize *R*^2^). Some examples are the Box and Cox (1964) family of transformations (see also Vélez et al., 2015), the alternating conditional expectation (ACE) method of Breiman and Friedman (1985), and Tibshirani's (1988) additive and variance stabilization (AVAS) approach. These transformations treat non-normality in ***e*** as a nuisance to be transformed away. Box-Cox transformations have been recommended as a method to address non-normality in linear regression (e.g., see Cohen et al., 2003; Osborne, 2010), and we clarify below that this transformation is more appropriate in the context of prediction.

Consider the well-known Box-Cox family of power transformations, which makes use of maximum likelihood estimation (MLE) to determine an optimal transformation. Suppose the linear model is modified to include a transformation parameter γ such that Equation (1) becomes

(2) $$\begin{array}{l} y_{i}^{\left(\gamma \right)}=\beta_{0}+\beta_{1}x_{1i}+\cdots +\beta_{K}x_{Ki}+\epsilon_{i}, \end{array}$$

where

$$\begin{array}{l} y^{\left(\gamma \right)}=\{\begin{array}{cc} \frac{y^{\gamma }-1}{\gamma } & \text{for}\gamma \neq 0\\ \log_{e}\left(y\right) & \text{for}\gamma =0. \end{array} \end{array}$$

Equation (2) is optimized such that estimates of the parameters of the model, (γ, ***β***), are determined by MLE under the constraint that the transformed DV, *y*^(γ)^, in Equation (2) follows a normal distribution. From Equation (1), results from OLS and MLE are identical; and maximizing the likelihood for Equation (1) is equivalent to maximizing *R*^2^ (Pek et al., 2016). The transformation determined by $\hat{\gamma }$ is said to normalize the residuals, ***e***. With normalized residuals, the assumption regarding the normality of ***ϵ*** is likely to be met. It follows then that results based on the transformed DV will be statistically more efficient relative to the original DV, be associated with more powerful NHSTs, and have tighter CIs and prediction intervals. We consider the Box-Cox approach an algorithmic transformation because the optimal estimated transformation parameter, $\hat{\gamma }$, frequently obtains parameter estimates in an inaccessible scale (see section 3.4.3 on reverse transformations).

The ACE (Breiman and Friedman, 1985) and AVAS (Tibshirani, 1988) methods are related to the Box-Cox transformation in that the linear model from Equations (1) and (2) is generalized to

(3) $$\begin{array}{l} g\left(y_{i}\right)=f_{1}\left(x_{1i}\right)+f_{2}\left(x_{2i}\right)+\cdots +f_{K}\left(x_{Ki}\right)+\epsilon_{i}, \end{array}$$

where *g*(·) and *f*_*k*_(·), for *k* = 1, ⋯ , *K*, are functions of the random variables associated with the observed data. Optimal transformations under ACE and AVAS are determined from the maximization of *R*^2^ associated with Equation (3). In general, ACE is more flexible than AVAS in terms of restrictions to the transformations afforded by *g*(·) and the *K* *f*_*k*_(·) functions. The ACE and AVAS are considered powerful fitting algorithms (e.g., see Harrell, 2015, p. 377) for prediction problems as they do not have statistical inferential measures. These algorithmic transformations are related to generalized additive mixture models (Baayen et al., 2017) and generalized additive models for location, scale, and shape (GAMLSS; Stasinopoulos et al., 2018).

#### 3.4.2. Parametric transformations

Parametric transformations are conducted to improve the interpretation of results such that the transformation via *g*(*y*_*i*_) obtains a more meaningful scale and *structure* of the data. Here, non-normality in ***e*** is informative and explicitly modeled via a chosen transformation. For instance, the natural logarithmic scale is used in studies of sound in decibels, the Richter scale for measuring intensity of earthquakes follows a base-10 logarithmic scale, and speed is the reciprocal of time. We highlight the example of logarithmic transformations because such a transformation links an additive model to a multiplicative model (e.g., see Gelman and Hill, 2007, p. 59). Examples on other parametric transformations are presented in Pek et al. (2017b).

Recall the linear model in Equation (1). Suppose that the DV is transformed such that the linear model becomes

(4) $$\begin{array}{l} \log\left(y_{i}\right)=\beta_{0}+\beta_{1}x_{1i}+\cdots +\beta_{K}x_{Ki}+\epsilon_{i}. \end{array}$$

Exponentiating both sides of Equation (4), in a reverse transformation, yields

(5) $$\begin{array}{ll} y_{i} & =e^{\beta_{0}+\beta_{1}x_{1i}+\cdots +\beta_{K}x_{Ki}+\epsilon_{i}}\\ ={\beta^{\prime }}_{0}{\beta^{\prime }}_{1}^{x_{1i}}\cdot {\beta^{\prime }}_{K}^{x_{Ki}}{\epsilon^{\prime }}_{i}, \end{array}$$

where $\beta_{0}^{\prime }=e^{\beta_{0}}$, $\beta_{k}^{\prime }=e^{\beta_{k}}$ for *k* = 1, ⋯ , *K*, and $\epsilon_{i}^{\prime }=e^{\epsilon_{i}}$. By taking the logarithm of the DV, the IVs enter the model multiplicatively in Equation (5) when the DV is expressed in its original scale (cf., Equation 1 where the IVs enter the model additively). Additionally, the errors, $\epsilon_{i}^{\prime }$, enter the model multiplicatively instead of additively. Given a logarithmic transformation, when ***ϵ*** is normally distributed, **ϵ′** will be non-normally distributed by definition. Taken together, parametric transformations such as the logarithmic transformation will change the scale of the original DV, as well as the functional form relating ***X*** to the original DV. Example 3 below illustrates how to interpret findings based on a multiplicative model stemming from a logarithmic transformation. Note that the log_*e*_ transformation is a special case in the Box-Cox family of transformations, but is characteristically distinct from algorithmic transformations in that it improves the interpretation of results.

#### 3.4.3. Reverse transformations

In our review of textbooks, reverse transformations were sometimes recommended (15%) to aid in the interpretation of parameter estimates from a linear model fit to transformed data. For instance, given that the DV underwent a natural logarithmic transformation (i.e., Equation 4, with base *e*), a parameter estimate of ${\hat{\beta }}_{1}=0.05$ is the expected conditional increase in log_*e*_(*y*) due to a one unit increase in *x*_1_. By reverse transforming $\hat{\beta_{1}}$ to obtain ${\hat{\beta }}_{1}^{\prime }=e^{0.05}\approx 1.05$, a one unit increase in *x*_1_ is associated with a 5% increase in the original DV; a two unit increase in *x*_1_ is associated with a 10% increase in the original DV. The estimated effect of 1.05, after reverse transformation, is not additive but multiplicative due to the link the logarithmic transformation has between additive and multiplicative models. Although not reported, applying a logarithmic transformation to data from Examples 1 and 2 will yield a geometric mean estimate after applying a reverse transformation. A more extensive illustration of the logarithmic transformation is presented in Example 3.

We raise caution against mechanistically applying reverse transformations to interpret parameter estimates. Reverse transformations may not necessarily aid in interpretation. Consider a Box-Cox transformation where γ = 0.5 such that $y^{\left(0.5\right)}=\frac{\sqrt{y}-1}{0.5}=2\sqrt{y}-2$, and this transformed DV is regressed onto an IV. Note that *y* cannot take on negative values. The linear model in Equation (2) becomes

$$\begin{array}{l} 2\sqrt{y_{i}}-2=\beta_{0}+\beta_{1}x_{1i}+\epsilon_{i}. \end{array}$$

Reverse transforming this equation obtains

$$\begin{array}{l} y_{i}=\left[1+\beta_{0}+0.25\beta_{0}^{2}\right]+\left[\beta_{1}+0.5\beta_{0}\beta_{1}\right]x_{1i}\\ \;+\left[0.25\beta_{1}^{2}\right]x_{1i}^{2}+\left[1+0.5\beta_{0}+0.5\beta_{1}x_{1i}+0.25\epsilon_{i}\right]\epsilon_{i}\\ =\beta_{0}^{\prime }+\beta_{1}^{\prime }x_{1i}+\beta_{2}^{\prime }x_{1i}^{2}+\epsilon_{i}^{\prime }. \end{array}$$

The Box-Cox transformation with γ = 0.5 results in an additive model with a quadratic relationship between the IV and DV. Clearly, the parameters in the transformed (i.e., β_0_ and β_1_) and original scale (which were reverse transformed; i.e., $\beta_{0}^{\prime }$, $\beta_{1}^{\prime }$ and $\beta_{2}^{\prime }$) are difficult to interpret. Complicating interpretation further, the error in the original scale, ***ϵ***′, is a complex function of the normally distributed error, ***ϵ***, the IV, *x*_1_, and the parameters of the model, ***β***. It is ill-advised to reverse transform parameter estimates and their inferential statistics (i.e., standard errors and CIs; e.g., see Bland and Altman, 1996) without consideration of how transformations can change the functional relationship between ***y*** and ***X*** as well as the structure of the errors, ***ϵ***′.

Reverse transformations to aid in the interpretation of predicted values, $\hat{y}$, have also been examined. Consider the Box-Cox transformation example above, where a predicted DV value is determined by $ŷ=\left[{\hat{\beta }}_{0}^{\prime }+{\hat{\beta }}_{1}^{\prime }x_{1}+{\hat{\beta }}_{1}^{\prime }x_{1}^{2}\right]$ at some chosen *x*_1_ value. Although more efficient, predicted values based on reverse transformations are known to be biased and inconsistent relative to their commensurate counterparts derived without transformations. Methods which can be generalized across all types of transformations, such as the smearing estimate (Duan, 1983), have been developed to address reverse transformation bias. Reverse transformation methods tied to a parametric distribution in the transformed scale, such as the third-order asymptotic method (Pek et al., 2017a) and the Cox method (Zhou and Gao, 1997), remain an active area of research.

#### 3.4.4. Example 3: income and occupational prestige

This example illustrates how the reviewed methods above are applied to a more complex linear model with continuous IVs (cf. Examples 1 and 2). In general, the CLT, HCCMs, bootstrap, and transformation methods can be readily applied to linear models with multiple categorical or continuous IVs. Winsorizing and trimming were developed within the context of mean comparisons and are special cases in robust regression. In general, robust regression employs M-estimators, often termed iterated re-weighted least squares (IRWS), where Huber (1964) weights and biweights (Beaton and Tukey, 1974) are special cases. The accompanying Supplemental Material provides SAS and R code showing how these methods are implemented in Example 3.

We present an example on 1971 Census of Canada data focusing on occupational prestige reported in Fox and Weisberg (2011) and Fox (2015). The data are of *N* = 102 types of occupations (blue collar; white collar; and professional, managerial, and technical), the average education of occupational incumbents in years, the average income of incumbents in 1971 dollars, the percentage of incumbents within the occupation who are women, and the Pineo-Porter prestige score for occupation. We fit a simple model focusing on the conditional relationship of percentage of women (*x*_1_) and prestige (*x*_2_) on income (*y*); education was excluded from the model because of its high correlation with prestige, *r* = 0.85. The linear model is

$$\begin{array}{l} y_{i}=\beta_{0}+\beta_{1}x_{1i}+\beta_{2}x_{2i}+\epsilon_{i}, \end{array}$$

where the IVs have been mean-centered such that the intercept (β_0_) is the expected income for occupations with mean levels of prestige (46.8, *range* = [14.8, 87.2]) and percentage of women incumbents (29.0%). Table 4 presents results of OLS regression (i.e., CLT). The model accounted for 64% of the variance in income, and incumbents of occupations at the mean of prestige and percentage of women are expected to earn $6798 on average. A 1% increase in women incumbents within the occupation is associated with a decrease of $48 in income, holding prestige constant. And, a 1 point increase in Pineo-Porter prestige scores is associated with a $166 increase in income, holding percentage of women constant.

**Table 4:** Percentage of women incumbents and prestige on income in *N* = 102 Canadian occupations.

**Method**	**Intercept**			**% Women**			**Prestige**			
	**${\hat{\beta }}_{0}$**	***S.E.***	***95% CI***	${\hat{\beta }}_{1}$	***S.E.***	***95% CI***	${\hat{\beta }}_{2}$	***S.E.***	***95% CI***	***R***^2^
CLT	6797.9	254.79	[6292.3, 7303.5]	–48.4	8.1	[–64.5, –32.3]	165.9	15.0	[136.1, 195.6]	0.64
HC0	6797.9	251.02	[6299.8, 7296.0]	–48.4	5.7	[–59.8, –37.0]	165.9	22.3	[121.7, 210.1]	0.64
HC1	6797.9	254.79	[6292.3, 7303.5]	–48.4	5.8	[–60.0, –36.8]	165.9	22.6	[121.0, 210.7]	0.64
HC2	6797.9	256.05	[6289.8, 7306.0]	–48.4	5.9	[–60.0, –36.7]	165.9	22.8	[120.6, 211.2]	0.64
HC3	6797.9	261.22	[6279.6, 7316.2]	–48.4	6.0	[–60.3, –36.5]	165.9	23.4	[119.4, 212.4]	0.64
percBS	6797.9	–	[6331.0, 7331.0]	–48.4	–	[–61.4, 37.3]	165.9	–	[123.0, 211.1]	0.64
Bca	6797.9	–	[6387.0, 7408.0]	–48.4	–	[–65.3, 38.8]	165.9	–	[129.8, 221.3]	0.64
Huber Weights	6517.5	131.34	[6260.0, 6774.9]	–42.8	4.2	[–51.0, –34.6]	134.1	7.7	[118.9, 149.2]	0.59
Biweight	6389.7	120.88	[6152.7, 6626.6]	–41.4	3.9	[–49.0, –33.8]	122.9	7.1	[109.0, 136.8]	0.59
Box-Cox ($\hat{\gamma }=0.25$)^†^	31.23	0.25	[30.74, 31.73]	–0.07	0.01	[–0.09, –0.05]	0.21	0.01	[0.18, 0.24]	0.76
log_e_	8.66	0.03	[8.60, 8.72]	–0.01	0.001	[–0.01, –0.01]	0.02	0.002	[0.02, 0.03]	0.74
reverse loge^‡^	5770.5	–	–	0.99	–	–	1.02	–	–

All estimated parameters are statistically significant at the 5% level. S.E., standard error; CI, confidence interval; CLT, central limit theorem; HC, heteroscedastic consistent method; percBS, percentile bootstrap; BCa, bias corrected and accelerated bootstrap. Results based on iterated re-weighted least squares (IRWLS) are an extension of Winsorizing and trimming in linear regression models, where Huber (1964) weights and the biweight (Beaton and Tukey, 1974) are special cases.

† S.E.s and CIs are liberal because they have not been corrected for estimating γ.

‡ *We caution against reverse transforming S.E.s and CIs because they are biased and statistically inconsistent, and do not present them here*.

**Figure 4:**
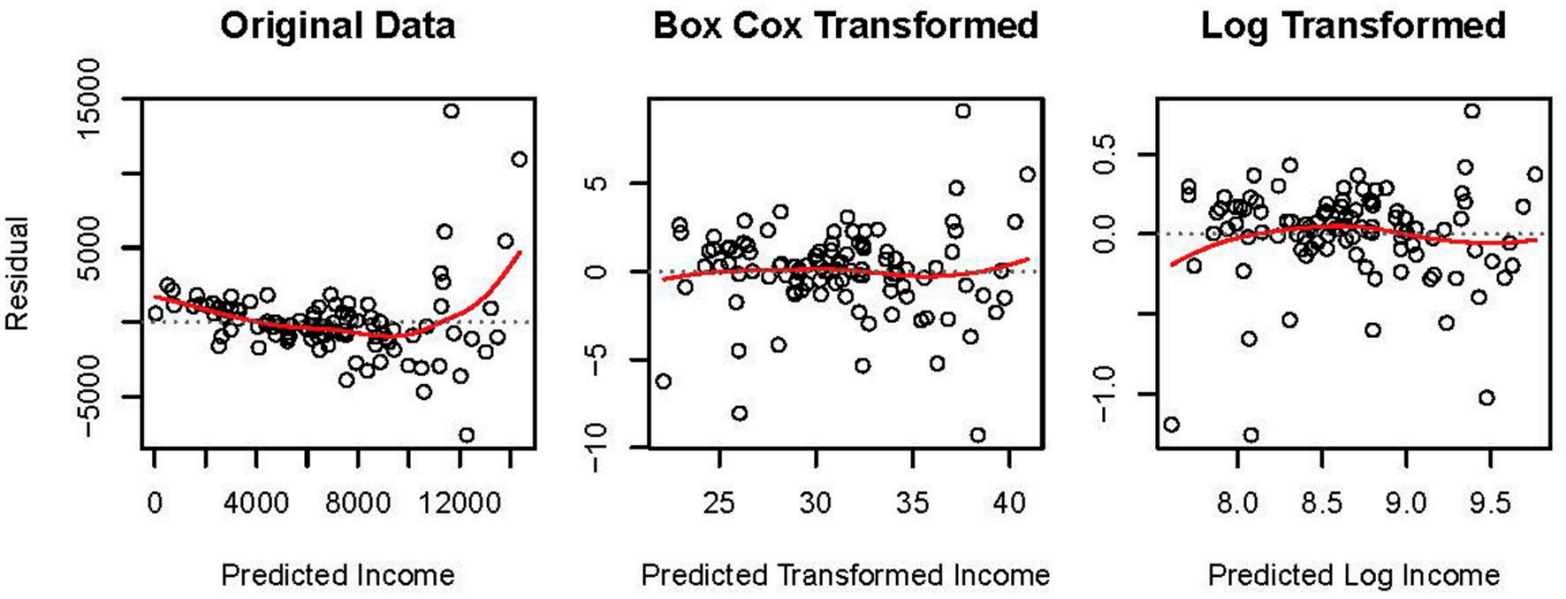
Residual by predicted plots for *N* = 102 Canadian occupations in 1971 where income, Box-Cox transformed income, and log-transformed income are regressed onto centered values of percentage of women incumbents and prestige scores. Spline curves are presented as solid lines overlaying the points.

The leftmost panel in Figure 4 presents the residual, ***e***, by predicted, $\hat{y}$, plot for the MLR model fit to original data. The residuals are not homoscedastic or normally distributed. A normal distribution is evident when points in such a plot form an ellipse. The larger spread of residuals at higher levels of predicted income in the plot of original data suggests heteroscedasticity. Additionally, the U-shaped spline overlaying the points suggests unmodeled non-linearity. Thus, alternative methods to OLS regression may be more appropriate for these data.

Table 4 presents results associated with alternative methods. Interpretations of the parameter estimates are identical across the CLT, HCCM, and bootstrap methods because the nature of the data remains unchanged resulting in unchanged parameters. All parameter estimates are significant at *p* < 0.001, and the results between these seven approaches differ superficially in terms of their standard error estimates and 95% CIs. Taken together, this group of methods answer the same research question regarding the conditional effects of women incumbents within and prestige scores for an occupation on income. Because sample size (*N* = 102) is not small, the observed differences among results derived from the CLT, HCCM, or bootstrap approaches are largely ignorable.

Methods following the tradition of Winsorizing and trimming yield different results from the CLT, HCCM, and bootstrap approaches because robust instead of arithmetic means are estimated. Recall that robust approaches are justified when extreme data points can be confidently regarded as outliers, which are removed or modified to limit data contamination. The linear model estimated with the M-estimators (e.g., Huber weights and biweights) account for a smaller amount of variance; *R*^2^ = 0.59 vs. *R*^2^ = 0.64. Additionally, the robust parameter estimates are smaller in magnitude due to the downweighting of extreme cases. As expected, the standard error estimates and 95% CIs for these robust estimates are also more efficient relative to the CLT, HCCM, and bootstrap methods. The validity of M-estimated parameters rests on confidence placed in the occurrence of data contamination which is manifest in outliers. The data were collected and maintained by the Census of Canada which is unlikely to have coding errors (Barnett and Lewis, 1998); however, the non-normality in the residuals may be indicative of contamination by an unknown non-target population giving rise to the observed heterogeneity.

Two transformations were applied to the data: the algorithmic Box-Cox transformation, and the parametric natural logarithmic transformation. Residual, ***e***, by predicted, $\hat{y}$, plots for these transformed data are presented in Figure 4. The transformations somewhat ameliorated the observed heteroscedasticity and non-linearity in the original data. The spread of the residuals toward higher levels of transformed income is less extreme relative to the original data. Additionally, the splines associated with the transformed data no longer suggest as strong a non-linear relationship as recovered from the original data. Comparing the central to the rightmost plot in Figure 4, the Box-Cox transformation linearized the data slightly more effectively compared to the natural log transformation. This observation is expected because the Box-Cox approach was developed to estimate values of γ via MLE which best linearizes the relationship between ***y***^(γ)^ and ***X*** while normalizing ***e***.

Table 4 also presents results from the Box-Cox transformation, the natural logarithmic transformation, and the reverse transformation of the natural logarithmic transformation. Reverse transformations for the Box-Cox approach did not aid in interpreting parameter estimates and are not reported. Compared to the other methods, these data transformation methods accounted for the most variance in the outcome. The Box-Cox approach had the largest *R*^2^ = 0.76 because $\hat{\gamma }$ was determined by maximizing *R*^2^, and was associated with more powerful tests relative to the other methods. However, the cost of this gain in efficiency is uninterpretable parameter estimates. Algorithmic transformations, which are extremely powerful tools for prediction, are not recommended for inference.

In contrast, reverse transforming parameter estimates obtained from the natural logarithmic transformation yielded intuitively appealing results. On average, occupations with mean levels of women incumbents and mean prestige ratings had an income of $5770. There was a negative conditional effect of the presence of women within an occupation on income. A 1% increase in female incumbents within an occupation is associated with a 1% decrease in income, holding prestige constant. For example, given an average income of $5770, an increase of 1% of women in the occupation would predict a decrease of income of $58 to 0.99 × 5770 = *$*5712, holding prestige constant. In contrast, prestige had a positive conditional effect on income; a 1 point increase in Pineo-Porter prestige scores is associated with a conditional 2% increase in the occupation's income. It is important to note that although reverse transformations of the parameter estimates are unbiased and sensible, reverse transformations of standard errors and CIs exhibit reverse transformation bias (e.g., see Pek et al., 2017a).

### 3.5. Other approaches

To this point, we have emphasized and illustrated how non-normality in OLS regression residuals can be taken into account by multiple approaches which remain within the linear modeling framework. Our review of undergraduate and graduate textbooks suggest other approaches which depart from the linear model (see Table 1). These approaches can be typically organized into rank-based non-parametric methods and non-linear models.

#### 3.5.1. Rank-based non-parametric methods

Rank-based methods analyze ranks derived from quantitative data. As such, the distribution of ranks no longer follow a parametric distribution (e.g., normality) but is obtained through permutation. Examples of rank-based non-parametric methods are the Wilcoxon rank-sum test (cf., two sample *t*-test), the Kruskal-Wallis test (cf., one-way ANOVA), the Wilcoxon signed-rank test (cf., paired sample *t*-test), and Spearman's correlation for ranked data (cf., Pearson's correlation). By transforming continuous data to ranks, the test of mean differences or linear relationships is replaced by tests of distributional differences and monotonic relationships, respectively. Interested readers are referred to the classic textbook by Siegel and Castellan (1988).

#### 3.5.2. Non-linear models

There are many types of non-linear models which are unified by their focus in modeling non-normal residuals, ***e***, observed from OLS regression. Non-normal errors can be modeled by specifying a non-linear relationship between ***y*** and ***X***, specifying a non-normal distribution for ***ϵ***, or both. For instance, non-linear regression analysis (Gallant, 1987) allows the functional form relating ***X*** to ***y*** to be non-linear. An example of a non-linear regression equation is

$$\begin{array}{l} y_{i}=\beta_{0}+\frac{1}{\beta_{1}x_{1i}+\beta_{2}}+\epsilon_{i}, \end{array}$$

where ***ϵ*** is an unobserved random error. Such a non-linear function can account for non-linearity in the residuals obtained from OLS regression. Alternatively, the form of the errors can be specified to be non-normal in non-linear models, while the functional relationship between ***X*** and ***y*** remains linear. For instance, the error term in Equation (1) can be specified to follow a Cauchy distribution with a non-centrality parameter. Nonlinear regression models often employ WLS and GLS estimators, or two-stage least squares.

Another well-known class of non-linear models is the generalized linear model (McCullagh and Nelder, 1989). Here non-normal parametric distributions are specified with a link function which results in a non-linear functional form as well as non-normal errors (e.g., poisson distribution for count data). Related to the generalized linear model is the GAMLSS (Stasinopoulos et al., 2018), which we referred to under the section on algorithmic transformation because it makes use of smoothing (i.e., non-parametric) techniques within a regression framework.

## 4. Summary and discussion

The linear model, and its special cases, tends to be the starting point of data analysis in the behavioral sciences. Often, researchers' foundational training in methodology does not extend beyond the linear model, inadvertently creating a pedagogical gap because of the ubiquity of observing non-normal residuals, ***e***, in practice. Non-normality in ***e*** *suggests* potential violation of the model's assumptions about ***ϵ***, which can result in inaccurate results (i.e., biased estimates and inaccurate inference). To answer the motivating questions, our review of undergraduate and graduate textbooks indicates that up to eight distinct approaches have been suggested (see Table 1), but in-depth coverage of these approaches was severely lacking. The reviewed textbooks are written from the frequentist perspective of probability, but Bayesian analogs to modeling non-normality are gaining traction (e.g., see Rubio and Genton, 2016).

We organized the identified methods into a new taxonomy according to three characteristics pertaining to whether methods continue within the linear modeling framework, whether the data are modified, and whether non-normality is considered a nuisance or informative (see Table 2). Focusing on methods pertaining to the linear model, we reviewed the motivations behind these approaches and illustrated that these methods can be grouped into three different sets of approaches which yield distinct results. In general, without changing the data while remaining within the linear modeling framework involves changing the estimator of the sampling distribution of $\hat{\beta }$. Changing the data results in changing the parameters of the linear model (e.g., arithmetic vs. geometric mean). Finally, the consideration of non-normality in ***e*** as a nuisance or as informative, from a theoretical perspective, would promote some methods over others. We forward that these considerations are pertinent to choosing a method to take into account non-normality, where a reasoned argument to justify their use is made (Abelson, 1995; cf., adjudicating the application of methods based on statistical significance).

In the first set of approaches, the CLT, HCCM, and bootstrap regard non-normality as a nuisance and circumvent the assumption of $\epsilon \sim MVN\left(0,\sigma^{2}I_{N}\right)$ with statistical theory (e.g., the CLT) or changing the estimator so as to relax assumptions about the form of the errors, ***ϵ*** (e.g., HCCM and bootstrap). When sample size *N* is large (e.g., Examples 2 and 3), the limited variability between results derived within this set of methods suggest that the linear model is aptly specified. Divergence across results from the CLT, HCCM, and bootstrap approaches could indicate sampling variability (e.g., Example 1) or that the model is misspecified. King and Roberts (2014) propose that HCCMs are useful for detecting model misspecification. In general, this group of methods assumes that all cases are valid and non-normality in ***e*** arises from misspecifying the distribution of ***ϵ***.

In the second set of robust approaches (e.g., Winsorizing and trimming), non-normality due to outliers is regarded as indicative of data contamination. Such data contamination is addressed by modifying or discarding extreme data points. In contrast to the first set of approaches, non-normality in ***e*** points to invalid data of cases, *i*, instead of model misspecification. Much information about the nature of the phenomenon under study, and the characteristics of valid data, is required to confidently identify and justify removing or recoding outliers. For this reason, we caution against the common practice of setting up strict data cleaning rules for trimming or Winsorizing data prior to their collection and exploration (e.g., Tabachnick and Fidell, 2012), and erroneously employing OLS estimation on the modified data. Here, the parameters of the linear model become robust analogs of the parameters estimated under CLT, HCCM, and the bootstrap. M-estimators are used to address dependency resulting from changing the data based on their rank order. Robust approaches imply that the data come from multiple populations: the target population in combination with other nuisance population(s).

Instead of employing robust approaches, mixture regression (e.g., see McLachlan and Peel, 2004) and quantile regression (Koenker and Bassett, 1978; Waldmann, 2018) are alternative methods which were developed to directly model population heterogeneity. In brief, mixture regression and quantile regression are considered semi-parametric or non-parametric methods, in contrast to the parametric method of MLR. In MLR, a single distribution (i.e., normal) is fit to the data. In mixture regression, a weighted sum of distributions (e.g., a mixture of several normal distributions) is fit to data; each distribution is typically interpreted as representing an unknown group, and the combination of several distributions create the observed heterogeneity. Alternatively, quantile regression involves fitting a model based on the quantiles of the observed data distribution instead of the quantiles of a parametric distribution (e.g., normal); different quantiles are often taken to represent different groups within the population.

The third and final set of methods involve data transformations. Algorithmic transformations are extremely powerful tools for statistical prediction which treat non-normal residuals as a nuisance (e.g., see ACE by Breiman and Friedman, 1985 or AVAS by Tibshirani, 1988). Conversely, parametric transformations (e.g., log(*y*)) treat non-normality of ***e*** as informative; a well-chosen parametric transformation can effectively address non-normality in ***e*** by changing the functional form relating the original DV to the IVs, as well as the structure of the errors, ***ϵ***′. Data transformations implicitly assume that the linear model is misspecified in relation to valid data. Additionally, reverse transformations are pertinent to interpreting parameter estimates and predicted values in the original scale of the data. Users should be aware of reverse transformation bias in inferential devices (i.e., standard error estimates and CIs; Pek et al., 2017a) and predicted values (Duan, 1983), which continue to be an active area of research. Because of the apparent simplicity of data transformations, this method was most often recommended in graduate textbooks (89%, see Table 1). Unfortunately, the complexities of uninterpretable scales and reverse transformation bias lacked emphasis in the reviewed textbooks, potentially encouraging their mispplication.

Data analysis is akin to conducting detective work (Tukey, 1969). Often, the linear model does not account for all the characteristics in the data, resulting in the ubiquity of observing non-normally distributed residuals, ***e***. Many methods have been developed to address different sources of misfit between data and model. These methods either assume error in the model or error in the data. Many different models and approaches can successfully disentangle the signal from the noise inherent in data. Choosing the most appropriate approach depends on statistical properties and, more importantly, theoretical assumptions about the data, the hypothesized functional relationship between ***y*** and ***X***, and assumptions about the structure of ***ϵ***. To attain mastery of these alternative approaches, which can address non-normal residuals from a linear model, requires a commitment to delve beyond what we have briefly reviewed here. We anticipate that our review, taxonomy, and examples provide a starting point for researchers intent on extending their knowledge in approaches developed to address non-normality from the perspective of the linear model.

## Author contributions

All authors contributed to the writing of the paper. JP and OW conducted the systematic review of textbooks. OW summarized the data from the systematic review. JP derived the taxonomy, identified, and analyzed empirical examples.

### Conflict of interest statement

The authors declare that the research was conducted in the absence of any commercial or financial relationships that could be construed as a potential conflict of interest.

## Footnotes

^1^The terminology of non-parametric transformations is adopted from Harrell (2015, p. 376), and the newly coined phrase “parametric transformations" follows in juxtaposition.
