# Supplementary material for: How to Address Non-normality: A Taxonomy of Approaches, Reviewed, and Illustrated
Source: Front Psychol. 2018 Nov 6;9:2104. doi: 10.3389/fpsyg.2018.02104 (PMC6232275; doi:10.3389/fpsyg.2018.02104)
Supplement: Supplementary file 1 [file Data_Sheet_1.pdf]

Supplemental Material

to accompany

Pek, J., Wong. O, & Wong, A. C. M. (2018). Addressing non-normality: A taxonomy of approaches, reviewed and illustrated. *Frontiers in Psychology*. *9*:2014. doi: 10.3389/fpsyg.2018.02104

SAS and R code, which accompany Example 3 on income and occupational prestige are provided below. Computations for heteroscedastic corrected covariance matrix (HCCM), robust regression using Huber Weights and biweight algorithms, and data transformations are conducted using SAS. The bootstrap approaches were implemented with R.

Heteroscedastic Corrected Covariance Matrix (HCCM)

```
proc reg data=prestige; *HC0;
  model income = women prestige/clb acov;
  output out=orig p=yhat r=resid;
run; quit;

proc reg data=prestige; *HC1;
  model income = women prestige/clb acov hccmethod=1;
  output out=orig p=yhat r=resid;
run; quit;

proc reg data=prestige; *HC2;
  model income = women prestige/clb acov hccmethod=2;
  output out=orig p=yhat r=resid;
run; quit;

proc reg data=prestige; *HC3;
  model income = women prestige/clb acov hccmethod=3;
  output out=orig p=yhat r=resid;
run; quit;
```

Bootstrap

```
library(boot)

fc <- function (formula,data,i){
  d <- data[i,]
  fit <- lm(formula, data=d)
  return(coef(fit))
  }

bs1 <- boot(data=prestige, statistic=fc, R=1000, formula = income~women + prestige)
boot.ci(bs1, type = c("norm", "basic", "perc", "bca"), index=1) #intercept
```

```
boot.ci(bs1, type = c("norm", "basic", "perc", "bca"), index=2) #women
boot.ci(bs1, type = c("norm", "basic", "perc", "bca"), index=3) #prestige
```

Robust Regression

```
proc robustreg data=prestige method=m (wf=huber); *m-estimation, huber weight;
  model income = women prestige;
  output out = t3 weight=wght;
run;

proc robustreg data=prestige method=m (wf=bisquare); *m-estimation, tukey’s bisquare;
  model income = women prestige;
  output out = t3 weight=wght;
run;
```

Data Transformation

```
proc transreg data=prestige test; *obtain estimate of gamma;
model BoxCox(income) = identity(women education prestige);
output out=test coefficients noscores;
run;
proc print data=test; *print results of estimates of gamma;
run;

data prestige; set prestige;
  bcincome = (income**.25 -1)/.25; *box-cox transform;
  lincome = log(income); *log-transform;
run;

proc reg data=prestige; *original data - CLT;
  model income = women prestige/clb;
  output out=orig p=yhat r=resid;
run; quit;

proc reg data=prestige; *box-cox transformed data;
  model bcincome = women prestige/ clb;
  output out=bc p=yhat r=resid;
run; quit;

proc reg data=prestige; *log-transformed data;
  model lincome = women prestige/ clb;
  output out=log p=yhat r=resid;
run; quit;
```
